# A hierarchical model of vision (HMAX) can also recognize speech

**DOI:** 10.1186/1471-2202-15-S1-P187

**Published:** 2014-07-21

**Authors:** Matthew J Roos, Michael Wolmetz, Mark A Chevillet

**Affiliations:** 1 Johns Hopkins University-Applied Physics Lab, Laurel, MD 20723, USA

## 

HMAX is a well-known computational model of visual recognition in cortex consisting of just two computational operations – a “template match” and non-linear pooling – alternating in a feedforward hierarchy in which receptive fields exhibit increasing specificity and invariance [1]. Interestingly, auditory recognition problems (such as speech recognition) share similar computational requirements, and recent work in auditory neuroscience suggests that auditory and visual cortex share similar anatomical and functional organization. Based on these similarities, we tested whether HMAX could support an auditory recognition task (specifically, word spotting).

To test HMAX on word spotting, recorded speech samples from the TIMIT corpus [2] were first converted into time-frequency spectrograms using a computational model of the auditory periphery [3]. These spectrograms were then split into 750 ms frames and input to a standard HMAX model [4]. Based on observed similarities between the receptive fields in primary auditory cortex (spectro-temporal receptive fields, or STRFs) and primary visual cortex (typically modeled as oriented Gabor filters), we used S1 filters identical to those used in vision [4]. Similarly, S2 “patches” were randomly selected from C1 representations of speech sounds drawn from an independent speech corpus. One vs. all linear support vector machines (SVMs) were then trained to discriminate frames that contain a target word from those that did not. These SVMs were then tested on a novel set of test sentences using a sliding frame approach (750 ms frame size, 20 ms step size). For each frame in a sentence, the SVM produced a distance from the hyperplane, and a threshold value was applied to produce a binary classification whether or not the target word was present in the sentence. When tested on target words that appeared in a fixed context (i.e. SA sentences in TIMIT), performance was highly robust, with ROC areas consistently above 0.9. When tested on target words that appeared in variable contexts (i.e., SI sentences in TIMIT), performance was somewhat decreased with ROC areas around 0.8. This decrease in performance is likely due to the inclusion of “clutter” (i.e., target irrelevant features) within the frame, also commonly observed when HMAX is applied to visual object recognition tasks [1].

These results are novel in that they provide support for the hypothesis that the simple computational framework implemented in HMAX – consisting of a feedforward hierarchy of only two alternating computational operations – may generalize beyond vision to support auditory recognition as well. It is possible that such a representation could give rise to stable neural encodings that are invariant to behaviorally irrelevant characteristics as seen in higher order visual and auditory cortices [5,6]. While it is likely that this auditory version of the HMAX model would benefit from the use of more auditory-specific filters based on STRF models [7], the Gabor features used here are largely compatible with previous computational models based on STRFs up to the level of primary auditory cortex [8]. Additional benefit may also be gained by learning sparse representations from natural sounds, at both the S1 and S2 levels [9].
